# Adult colonic intussusception secondary to caecal malignancy: diagnostic and temporary therapeutic role of contrast enema

**DOI:** 10.1093/jscr/rjag499

**Published:** 2026-06-25

**Authors:** Sumra Irum, Mikail Kale, Anders L Elgborn, Per Helligsø

**Affiliations:** Department of Radiology, University Hospital of Southern Denmark, Kresten Philipsens Vej 15, 6200 Aabenraa, Denmark; Department of Surgery, University Hospital of Southern Denmark, Kresten Philipsens Vej 15, 6200 Aabenraa, Denmark; Department of Surgery, University Hospital of Southern Denmark, Kresten Philipsens Vej 15, 6200 Aabenraa, Denmark; Department of Surgery, University Hospital of Southern Denmark, Kresten Philipsens Vej 15, 6200 Aabenraa, Denmark

**Keywords:** adult intussusception, colonic intussusception, caecal adenocarcinoma, contrast enema, computed tomography, bowel obstruction

## Abstract

Adult colonic intussusception is rare and often associated with malignancy; however, the underlying lead point may remain occult on initial imaging. We report a 75-year-old woman presenting with two weeks of right lower quadrant pain, initially managed as a urinary tract infection. Computed tomography (CT) demonstrated colonic intussusception without bowel obstruction or a clearly identifiable lead point. Given the patient’s stable condition, non-operative reduction was attempted. A CT-guided water-soluble contrast enema resulted in resolution of the intussusception and revealed a caecal mass not identified on initial imaging. The patient subsequently underwent planned surgical resection for caecal adenocarcinoma. This case highlights the diagnostic value and potential temporary therapeutic role of contrast enema in selected adult patients, facilitating identification of an underlying pathology without delaying definitive treatment.

## Introduction

Adult colonic intussusception is rare and frequently associated with an underlying malignancy; however, the causative lead point may remain obscured on initial imaging. Adult intussusception accounts for ~5% of all intussusceptions and 1%–5% of bowel obstructions in adults [1, 2]. Unlike paediatric disease, where non-operative reduction is standard, adult intussusception is usually secondary to a pathological lead point, most frequently a neoplasm, and is therefore managed surgically [3, 4].

Non-operative reduction techniques such as contrast enema are rarely used in adults due to concerns regarding perforation, tumour dissemination, and delay of definitive oncological treatment [1, 3]. Computed tomography (CT) is the primary diagnostic modality in adults; however, the underlying lead point may remain obscured within the intussuscepted segment on initial imaging, limiting accurate preoperative characterization [5].

We present a case in which CT-guided contrast enema served as a selective diagnostic and temporary therapeutic tool, allowing reduction of the intussusception and subsequent identification of an underlying caecal malignancy.

## Case report

A 75-year-old woman with a history of bilateral breast cancer, ischaemic stroke (on long-term clopidogrel therapy), previous appendicectomy, and hysterectomy with bilateral salpingo-oophorectomy presented with a 2-week history of right lower quadrant abdominal pain. The pain was constant with intermittent exacerbations and associated with nausea and one episode of vomiting.

At initial presentation, the symptoms were attributed to a urinary tract infection based on a positive urine dipstick test, and she was treated with pivmecillinam without clinical improvement. Two weeks later, she was readmitted, and contrast-enhanced CT of the abdomen was performed.

The examination demonstrated colonic intussusception without signs of bowel obstruction or definite malignancy. Importantly, no clear lead point was identified on the initial CT examination (Fig. 1). Given the absence of obstruction and the patient’s stable clinical condition, contrast enema reduction was considered.

**Figure 1:**
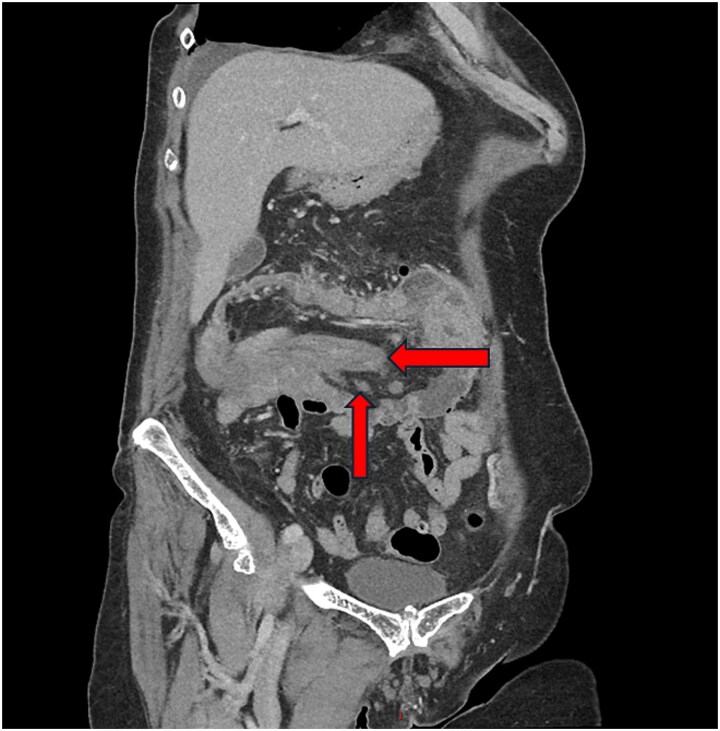
Coronal contrast-enhanced CT image demonstrating colonic intussusception (arrows) with a characteristic bowel-within-bowel configuration, including invaginated mesenteric fat and vessels.

Following multidisciplinary discussion, contrast enema was performed using 2 L of 5% water-soluble contrast administered per rectum, with free passage to the caecum. Follow-up CT demonstrated resolution of the intussusception and revealed a caecal mass measuring 63 × 60 × 44 mm (Fig. 2), enabling identification of the previously occult pathological lead point.

**Figure 2:**
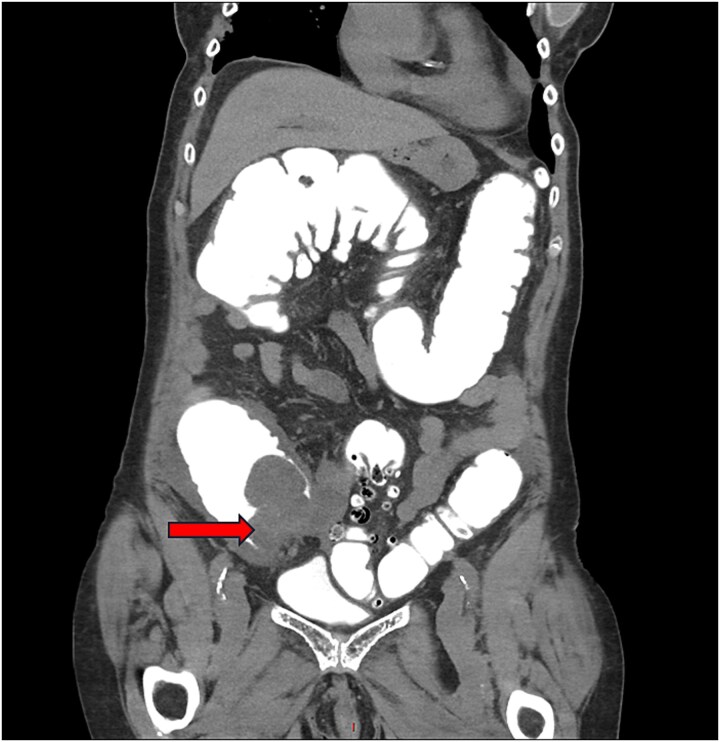
Coronal CT image following contrast enema demonstrating resolution of the intussusception and a caecal mass (arrow) outlined by retrograde contrast.

The patient was discharged in good general condition and referred via an expedited cancer diagnostic pathway. She underwent laparoscopic right hemicolectomy for a biopsy-proven caecal adenocarcinoma with primary ileocolic anastomosis. There were no intraoperative complications and no evidence of metastatic disease.

Histopathological examination demonstrated a 90-mm caecal adenocarcinoma with invasion through the muscularis propria. Fourteen lymph nodes were negative for metastasis, and resection margins were free (pT3N0M0). The postoperative course was uncomplicated.

## Discussion

Adult intussusception differs fundamentally from paediatric disease in aetiology and management. Most adult cases are secondary to benign or malignant structural lesions [1, 2], with malignancy being particularly common in colonic intussusception [1, 3, 4]. This finding underlies the recommendation for primary surgical resection without prior reduction in most adult cases [3, 4]. Several case reports have similarly described adult colonic or ileocolic intussusception secondary to colonic adenocarcinoma, highlighting the importance of considering an underlying malignancy as the lead point [6, 7].

Non-operative reduction remains uncommon and controversial due to concerns regarding perforation, tumour dissemination, and delay in definitive oncological management [1]. However, this approach assumes that the underlying pathology is adequately characterized on imaging. In practice, CT may fail to identify the lead point, as it can remain obscured within the intussuscepted bowel and surrounding oedematous mesentery [5].

This case highlights that limitation. The underlying caecal malignancy was not identifiable on the initial CT examination but became evident only after resolution of the intussusception. In this setting, contrast enema provided additional diagnostic clarity and enabled identification of the underlying pathology.

Whilst non-operative reduction should not be routinely performed in adults, carefully selected hemodynamically stable patients without signs of obstruction, ischemia, or perforation may benefit from an individualized approach [1–3]. In this case, reduction did not delay definitive surgical management but facilitated diagnosis and planning.

Thus, contrast enema may serve as a selective diagnostic adjunct in adult intussusception when the lead point is not clearly identified, rather than as an alternative to surgery.

## Conclusion

Adult colonic intussusception is rare and frequently associated with underlying malignancy. Surgical resection remains the definitive treatment. CT-guided contrast enema may serve as a selective diagnostic and temporary therapeutic adjunct in carefully chosen stable patients when the lead point is not clearly identified on initial imaging, without delaying definitive surgical management.
